# Hot topics and trends in acupuncture for herpes zoster from 2015 to 2025: a bibliometric and knowledge graph analysis

**DOI:** 10.3389/fmed.2026.1748878

**Published:** 2026-03-11

**Authors:** Zhanhong Xi, Lubing Zhu, Xiaolian Peng

**Affiliations:** 1 Department of General Practice, Sunqiao Community Health Service Center, Shanghai, China; 2 Department of Dermatology, Zhongshan Hospital, Fudan University, Shanghai, China; 3 Department of General Practice, Huinan Community Health Service Center, Shanghai, China

**Keywords:** acupuncture, bibliometric analysis, CiteSpace, herpes zoster, postherpetic neuralgia, VOSviewer

## Abstract

**Background:**

This study aimed to systematically and comprehensively analyze global research trends, knowledge structure, and emerging hotspots in acupuncture for herpes zoster (HZ) and postherpetic neuralgia (PHN).

**Methods:**

Publications from January 1, 2015, to October 1, 2025 were retrieved from six major databases, including CNKI, Wanfang, Weipu, Web of Science Core Collection, PubMed, and Scopus. CiteSpace and VOSviewer were employed to perform co-authorship, co-citation, and keyword analyses, visualizing research collaborations, thematic evolution, and knowledge networks.

**Results:**

A total of 2,309 publications (2,165 Chinese and 144 English) were included. In the Chinese databases, publication output peaked in 2018, while English-language publications steadily increased, surpassing 20 per year after 2022. All Chinese studies originated in China, whereas 21 countries contributed to the English-language literature, with China, the United States, Australia, and South Korea as the top contributors. Zhejiang Chinese Medical University, Guangzhou University of Chinese Medicine, and Chengdu University of Traditional Chinese Medicine were the most productive institutions. Co-authorship and citation analyses identified core authors and influential journals, including PAIN, Evidence-Based Complementary and Alternative Medicine, and J Pain Res. Keyword co-occurrence and clustering analyses revealed frequent topics in Chinese publications, such as PHN, acupuncture and cupping, fire needle, TCM, and Jiaji point, whereas English publications frequently addressed HZ, PHN, neuropathic pain, management, and acupuncture. Timeline and citation-burst analyses indicated shifts in research focus over time, with early studies emphasizing traditional techniques and clinical efficacy, and later studies increasingly addressing mechanisms, patient-reported outcomes, and systematic evidence synthesis.

**Conclusion:**

Global research on acupuncture for HZ and PHN has increasingly shifted toward precision and evidence-based approaches. Comparative analysis indicates that Chinese literature primarily emphasizes clinical applications and traditional Chinese medicine techniques, whereas international studies focus more on methodological rigor and mechanistic evidence, underscoring the need for strengthened international collaboration and high-quality multicenter trials to advance the field.

## 1 Introduction

Herpes zoster (HZ), commonly known as shingles, is a painful skin disease caused by reactivation of latent varicella-zoster virus (VZV) in sensory ganglia (1). It predominantly affects older adults and immunocompromised individuals, with a lifetime incidence estimated at 20–30% (2). Postherpetic neuralgia (PHN), the most common complication of HZ, is characterized by persistent neuropathic pain lasting months to years, significantly impairing quality of life and imposing substantial psychological and economic burdens (3, 4). Conventional antiviral and analgesic therapies, while partially effective, are often limited by adverse effects, incomplete pain relief, or the risk of drug dependence, particularly in elderly patients (5–7). Guidelines from the CDC and recent NEJM reviews emphasize the use of gabapentin, pregabalin, and tricyclic antidepressants for PHN, but highlight challenges such as dose-limiting side effects, renal considerations, and suboptimal pain control in a substantial subset of patients (6, 8). These limitations have stimulated growing interest in complementary and alternative therapies for safe and effective management of HZ-associated pain.

Acupuncture, a core component of traditional Chinese medicine (TCM), has been extensively applied to treat pain disorders, including neuropathic pain, musculoskeletal pain, and PHN (9, 10). Clinical evidence indicates that acupuncture can reduce pain intensity, improve sleep quality, and decrease the need for analgesic medications in patients with HZ (11). Various acupuncture modalities, such as electroacupuncture, warm acupuncture, and auricular acupuncture, have been explored, underscoring its therapeutic versatility (9). Recent neuroimaging and electrophysiological studies have provided insights into the central mechanisms of acupuncture analgesia, including modulation of pain-related brain networks and regulation of inflammatory pathways (12, 13). Despite these advances, a comprehensive assessment of global research trends, knowledge structure, and emerging topics in acupuncture for HZ remains lacking.

Bibliometric analysis, a quantitative method employing mathematical and statistical tools, enables systematic evaluation of publication patterns, research collaborations, and scientific trends within a given field. Bibliometric tools such as CiteSpace and VOSviewer have been successfully applied to map research landscapes in related fields of acupuncture, including stroke (14), demonstrating their utility in identifying collaborative networks, research hotspots, and thematic evolution. CiteSpace and VOSviewer are widely used software platforms for constructing knowledge maps and visualizing bibliometric networks (15). Through analysis of co-authorship, co-citation, and keyword co-occurrence, bibliometric approaches can identify research hotspots, influential authors and institutions, and the temporal evolution of scientific domains.

In this study, we conducted a bibliometric and knowledge graph analysis of literature investigating acupuncture for HZ in recent years. Unlike previous reviews or bibliometric studies that focused solely on English-language publications, this study systematically integrates both Chinese and English literature, allowing for a comparative analysis of domestic and international research trends. This approach highlights potential gaps in cross-language dissemination, identifies key contributors in different linguistic domains, and provides a more comprehensive understanding of global research patterns. Our objectives were to provide a comprehensive overview of the research landscape, identify key contributors and influential studies, and explore emerging topics and trends to inform future research directions in this field.

## 2 Materials and methods

### 2.1 Data sources and search strategy

A bibliometric analysis was conducted to investigate research trends and emerging topics in articles and reviews on acupuncture for HZ, covering publications from January 1, 2015, to October 1, 2025. Data were retrieved from both Chinese and English databases. The Chinese literature search was performed in the China National Knowledge Infrastructure (CNKI), Weipu, and WanFang databases, while the English literature search was conducted in the Web of Science Core Collection (WoSCC), PubMed, and Scopus.

Search strategies were developed according to the specific search rules and field tags of each database. In WoSCC, the Topic Search (TS) field was used with the following query: TS = [(acupuncture OR electroacupuncture OR moxibustion) AND (herpes zoster OR shingles OR postherpetic neuralgia)].

For PubMed, searches were conducted using a combination of Medical Subject Headings (MeSH) terms and free-text terms in the Title/Abstract fields: ((“Acupuncture Therapy” [MeSH] OR acupuncture [Title/Abstract] OR electroacupuncture [Title/Abstract] OR moxibustion [Title/Abstract]) AND (“Herpes Zoster” [MeSH] OR herpes zoster [Title/Abstract] OR shingles [Title/Abstract] OR postherpetic neuralgia [Title/Abstract])).

For Scopus, searches were performed using the TITLE-ABS-KEY field: TITLE-ABS-KEY ((acupuncture OR electroacupuncture OR moxibustion) AND (herpes zoster OR shingles OR postherpetic neuralgia)).

For the Chinese databases (CNKI, Wanfang, and Weipu), searches were conducted using subject terms and keywords corresponding to acupuncture interventions (acupuncture, electroacupuncture, and moxibustion) combined with disease-related terms (herpes zoster, shingles, and postherpetic neuralgia). The searches were performed in Chinese within the title, keywords, and abstract fields according to each database’s search settings.

### 2.2 Inclusion criteria

Studies were included if they met the following criteria:

1. Studies published between January 1, 2015, and October 1, 2025;
2. Studies available in full-text or with an abstract;
3. Studies published in either Chinese or English language.

### 2.3 Exclusion criteria

Studies were excluded if they met any of the following conditions:

1. Duplicate or repeatedly published studies;
2. Publications not in the form of research articles or reviews, including conference abstracts, case reports, reports, notices, letters, or editorial materials;
3. Studies unrelated to HZ, shingles, or PHN;
4. Records with incomplete bibliographic information, such as missing publication year, author names, affiliations, or keywords.

### 2.4 Study selection process

After exporting the retrieved literature into NoteExpress 3.5, duplicate records were removed. An initial screening was conducted independently by two reviewers, who assessed titles, abstracts, and keywords to identify potentially relevant studies. For records without abstracts, full texts were reviewed. The results of the initial screening were cross-checked, and studies passing this stage proceeded to a second round of full-text review. In cases of disagreement between the two reviewers, a third reviewer was consulted to make the final decision regarding inclusion.

**Figure 1:**
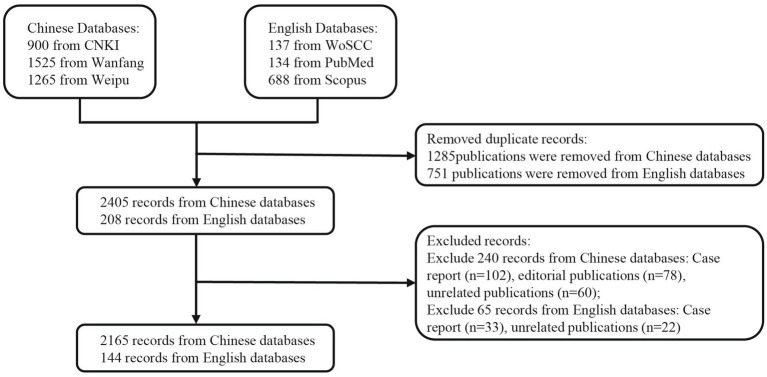
Flowchart of literature screening.

The study selection process is illustrated in Figure 1.

### 2.5 Data analysis

Descriptive statistical analyses were first performed using Excel 2021 on the exported database records, including annual publication counts and journal-wise publication volumes, to provide an overview of research output and distribution. Visual bibliometric analyses were subsequently conducted using CiteSpace (version 6.1. R6; Drexel University, Chaomei Chen) and VOSviewer (version 1.6.18; Leiden University, van Eck and Waltman) (16, 17).

CiteSpace is a Java-based visualization tool designed to explore the intellectual structure and dynamic evolution of scientific knowledge through co-citation, burst detection, and clustering analyses (16). It integrates data mining and knowledge mapping techniques to identify research hotspots, emerging topics, and collaborative relationships. VOSviewer (17), a scientometric network analysis software, provides network, overlay, and density visualizations that illustrate relationships among countries, institutions, journals, authors, keywords, and cited references. In these maps, node size reflects publication or citation frequency, while link thickness and distance indicate the strength of association; different colors represent clusters or temporal trends.

To gain an in-depth understanding of global research trends, collaboration patterns, and emerging hotspots in the field of acupuncture for HZ, CiteSpace and VOSviewer were jointly employed for comprehensive bibliometric analysis. These tools were used to generate visual knowledge maps and to analyze data on national and institutional cooperation, journals and co-cited journals, authors and co-authorship networks, as well as keyword co-occurrence and emerging terms. Through these analyses, this study aimed to elucidate the current research landscape, intellectual structure, and developmental trajectory of the field, thereby providing a valuable reference for future basic and clinical research.

## 3 Results

### 3.1 Analysis of publication trends

**Figure 2:**
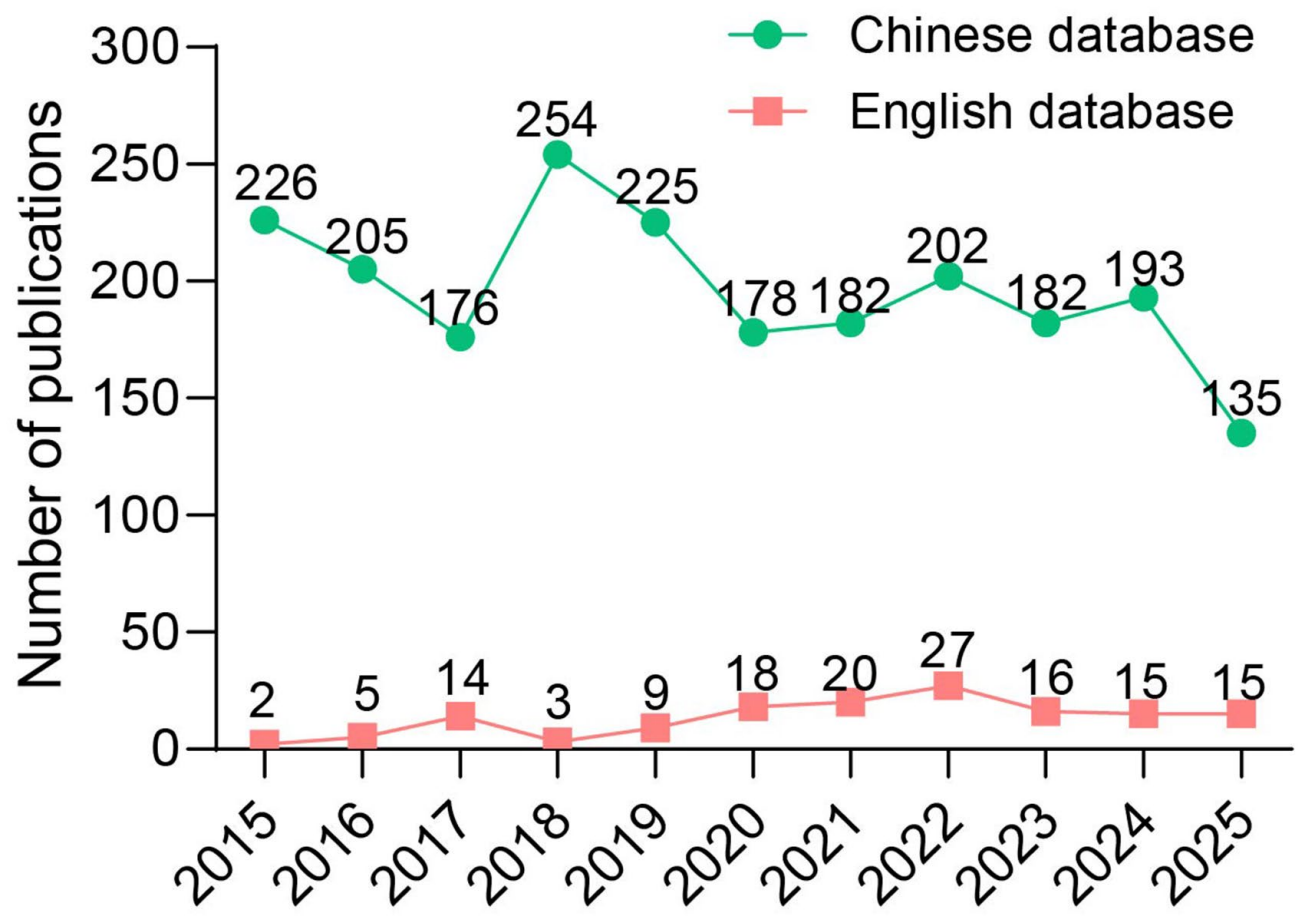
Annual publications on acupuncture for HZ from 2015 to 2025.

**Figure 3:**
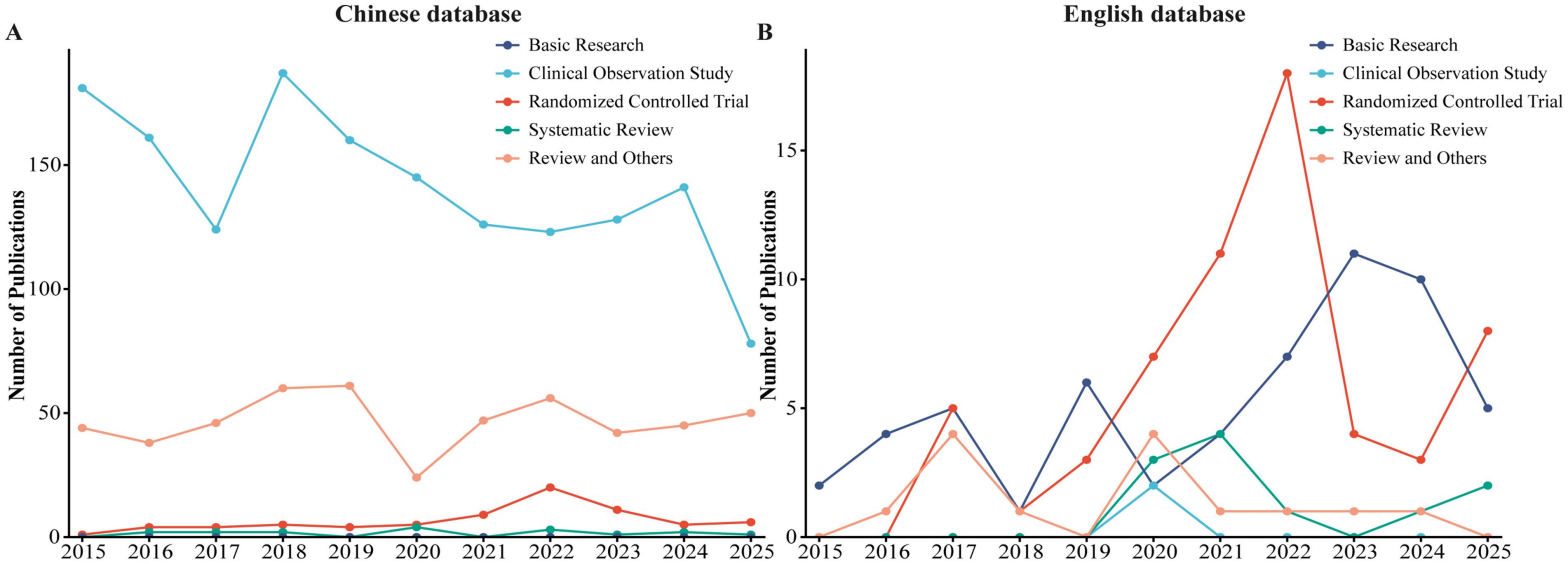
Evolution of study types in acupuncture for herpes zoster research (2015–2025). **(A)** Chinese-language publications. **(B)** English-language publications. Studies were categorized into five types: basic research (dark blue), clinical observation study (light blue), randomized controlled trial (red), systematic review (green), and review and others (orange).

Based on the predefined search strategy, 900, 1,525, and 1,265 records were retrieved from CNKI, Wanfang, and Weipu databases, respectively, while 137, 134, and 688 records were obtained from WoSCC, PubMed, and Scopus, respectively. After removing duplicates, 1,285 and 751 publications were excluded from the Chinese and English databases, respectively. Further screening excluded case reports, editorials, and irrelevant studies, resulting in a final inclusion of 2,165 Chinese and 144 English publications (Figure 1). As illustrated in Figure 2, the number of publications in the Chinese databases peaked in 2018, followed by minor fluctuations in subsequent years. In contrast, the English literature demonstrated a steady upward trajectory, surpassing 20 publications for the first time in 2022 and maintaining a relatively stable trend thereafter. To further examine the evolution of research paradigms, the included studies were categorized by type and their annual distribution was analyzed (Figure 3). Chinese-language publications were overwhelmingly dominated by clinical observation studies throughout the study period, with randomized controlled trials increasing notably after 2021. In contrast, English-language publications exhibited sustained high output in basic research and a marked rise in randomized controlled trials after 2019, peaking in 2025.

### 3.2 Distribution of countries

**Figure 4:**
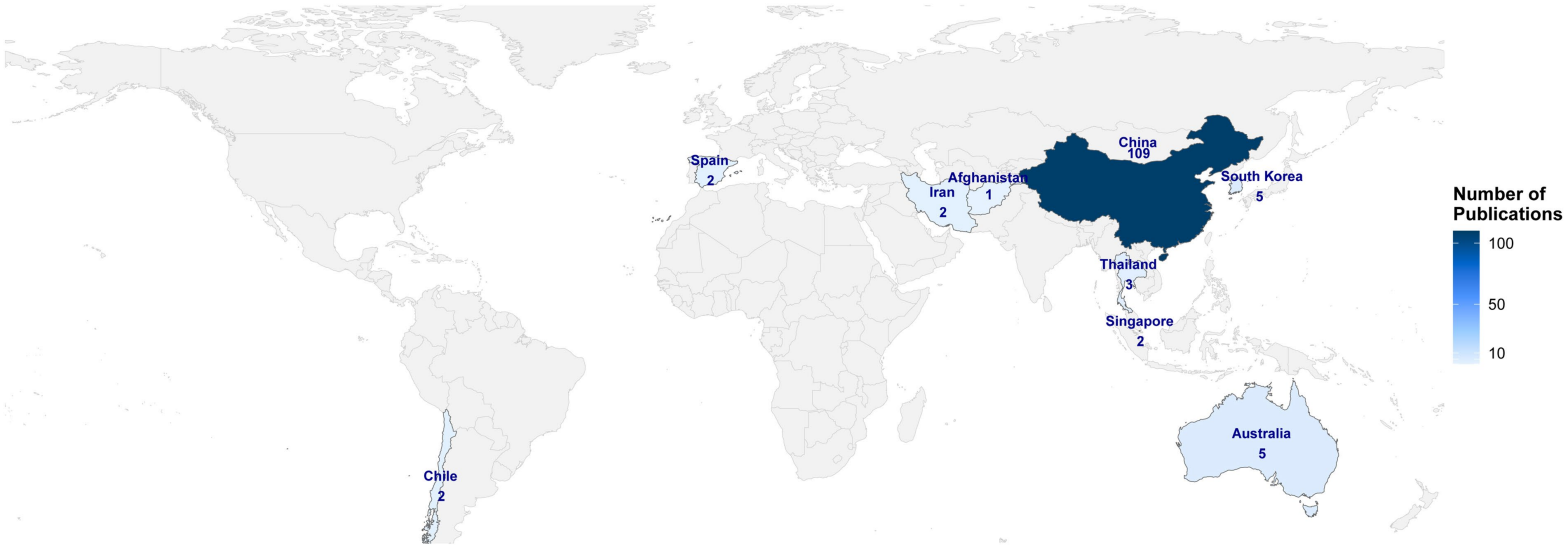
Geographical distribution of publications on acupuncture for herpes zoster by country. Choropleth world map showing the number of publications across contributing countries. Color intensity corresponds to publication volume, with darker shades indicating higher output.

**Figure 5:**
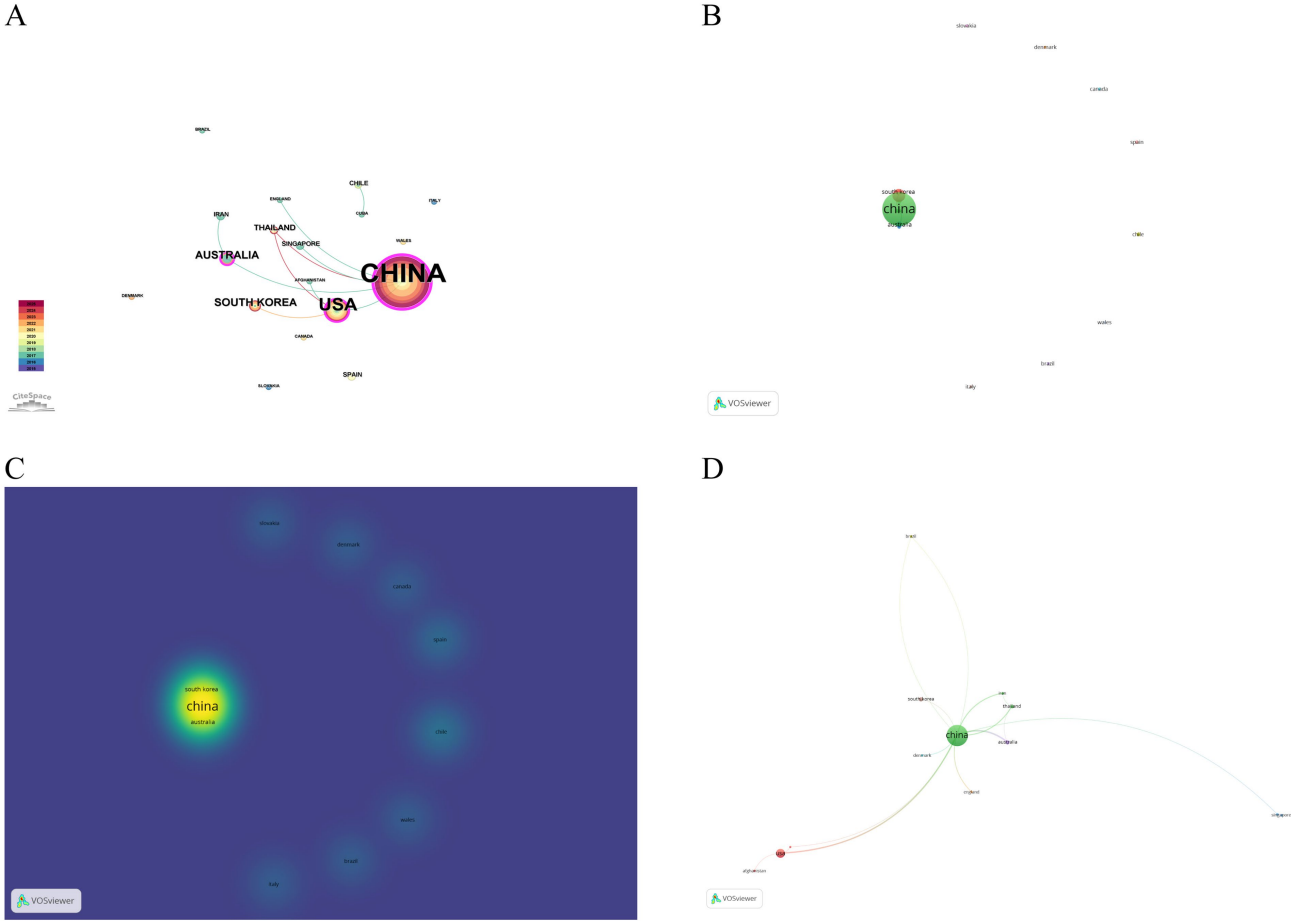
Country-level collaboration and citation analysis of acupuncture research on HZ from English databases. **(A)** Collaboration network of countries. **(B)** Overlay visualization of country collaborations. **(C)** Density visualization of country collaborations. **(D)** Overlay visualization of cited countries.

The Chinese-language literature is entirely domestically produced, indicating limited international dissemination within this linguistic domain. Therefore, no further country-level analysis was performed for these sources. Accordingly, the following results are based on publications indexed in English databases. The geographical distribution of publications by country is illustrated in Figure 4, which demonstrates the global research output and highlights the leading contributing nations. Country-level co-occurrence analysis conducted using CiteSpace revealed that 21 countries contributed to research on acupuncture for HZ in recent years (Figure 5A). China produced the highest number of publications (*n* = 109), followed by the United States (*n* = 19), Australia (*n* = 5), and South Korea (*n* = 5) (Table 1). In terms of betweenness centrality, which reflects the degree of international collaboration, China ranked first (0.43), followed by the United States (0.18) and Australia (0.17). Network and density visualizations generated by VOSviewer (Figures 5B,C) highlighted several prominent collaboration clusters, with China and the United States forming the central hubs of global cooperation. Citation network analysis (Figure 5D) further identified China and the United States as the most influential countries in this research domain, as evidenced by higher citation counts and stronger collaborative link strength.

**Table 1:** Top 10 countries contributing to publications on acupuncture for HZ based on English databases.

Rank	Countries	Publications	Centrality
1	China	109	0.43
2	United States	19	0.18
3	Australia	5	0.17
4	South Korea	5	0
5	Thailand	3	0
6	Chile	2	0
7	Iran	2	0
8	Singapore	2	0
9	Spain	2	0
10	Afghanistan	1	0

### 3.3 Distribution of institutions

**Figure 6:**
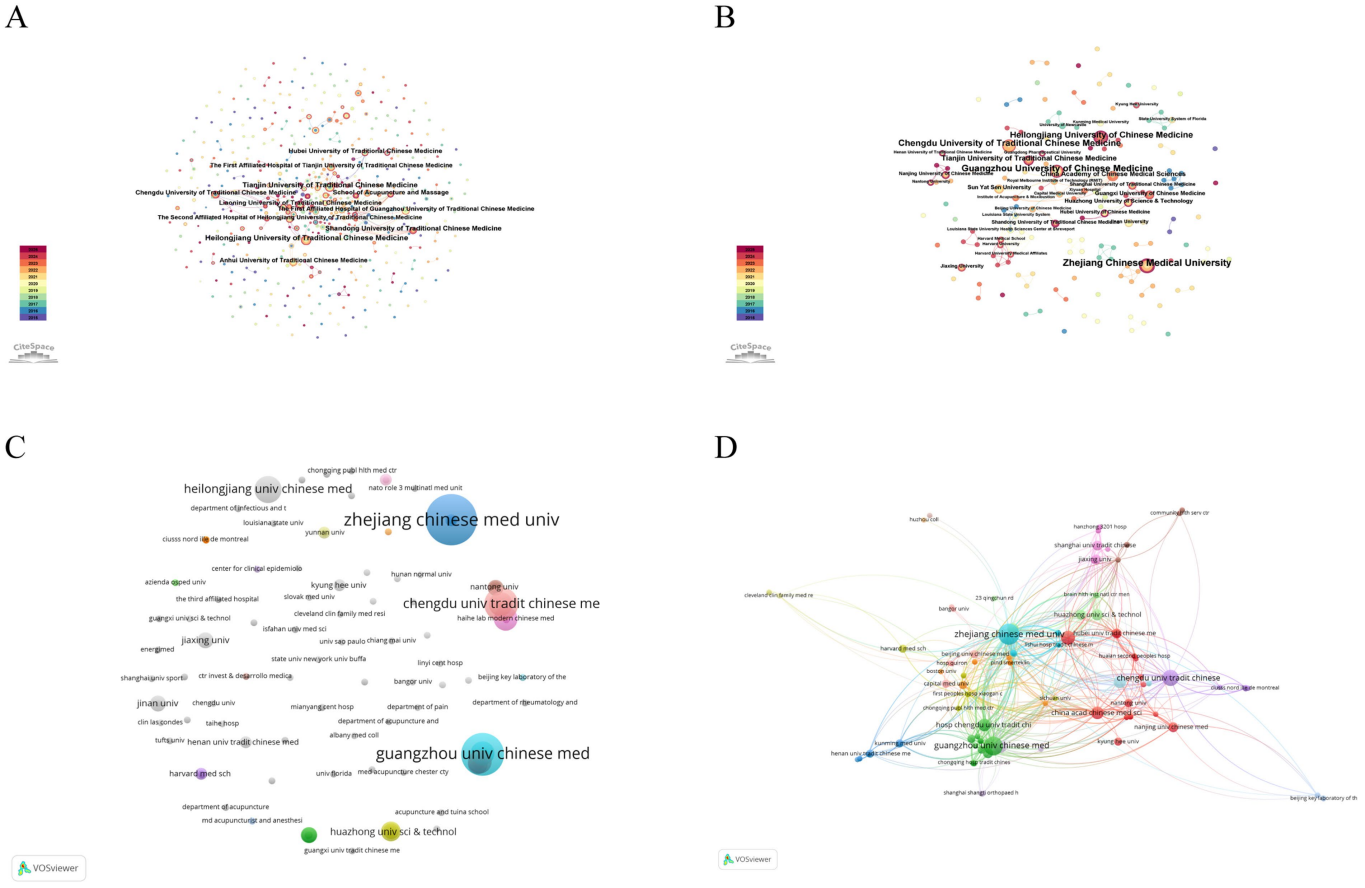
Institutional collaboration and citation analysis of acupuncture research on HZ. **(A)** Collaboration network of institutions from the Chinese databases. **(B)** Collaboration network of institutions from the English databases. **(C)** Overlay visualization of institutions from the English databases. **(D)** Overlay visualization of cited institutions from the English databases.

The institutional distribution of publications is illustrated in Figures 6A–D. In the Chinese database (Figure 6A), the leading institutions were Heilongjiang University of Traditional Chinese Medicine (41 publications, centrality = 0.02), Tianjin University of Traditional Chinese Medicine (37, 0.02), and Shandong University of Traditional Chinese Medicine (29, 0.02), followed by Hubei University of Traditional Chinese Medicine and the School of Acupuncture and Massage (26 each). In the English database (Figure 6B), Zhejiang Chinese Medical University produced the largest number of publications (*n* = 13), followed by Guangzhou University of Chinese Medicine (*n* = 11) and Chengdu University of Traditional Chinese Medicine (*n* = 10), with Chinese institutions collectively contributing the majority of outputs (Table 2). Co-authorship and citation network analyses performed using VOSviewer (Figures 6C,D) further identified Zhejiang Chinese Medical University (14 publications, 82 citations, total link strength = 188), Guangzhou University of Chinese Medicine (11, 97, 96), and Chengdu University of Traditional Chinese Medicine (8, 58, 63) as the most influential institutions, indicating their central positions and substantial academic impact within this research field (Table 3).

**Table 2:** Top 10 institutions publishing research on acupuncture for HZ in Chinese and English databases.

Rank	Chinese Database			English Database		
	Institution	Publications	Centrality	Institution	Publications	Centrality
1	Heilongjiang University of Traditional Chinese Medicine	41	0.02	Zhejiang Chinese Medical University	13	0
2	Tianjin University of Traditional Chinese Medicine	37	0.02	Guangzhou University of Chinese Medicine	11	0.01
3	Shandong University of Traditional Chinese Medicine	29	0.02	Chengdu University of Traditional Chinese Medicine	10	0
4	Hubei University of Traditional Chinese Medicine	26	0.01	Heilongjiang University of Chinese Medicine	9	0
5	School of Acupuncture and Massage	26	0.03	Tianjin University of Traditional Chinese Medicine	6	0
6	Liaoning University of Traditional Chinese Medicine	25	0.01	China Academy of Chinese Medical Sciences	5	0.01
7	Chengdu University of Traditional Chinese Medicine	23	0.02	Huazhong University of Science & Technology	4	0
8	The First Affiliated Hospital of Tianjin University of Traditional Chinese Medicine	22	0	Guangxi University of Chinese Medicine	4	0
9	The First Affiliated Hospital of Guangzhou University of Traditional Chinese Medicine	22	0.01	Sun Yat-sen University	4	0
10	Anhui University of Traditional Chinese Medicine	22	0.02	Shanghai University of Traditional Chinese Medicine	3	0

**Table 3:** Top 10 institutions in citation networks for acupuncture research on HZ based on English databases.

Rank	Institution	Publications	Citations	Total link strength
1	Zhejiang Chinese Medical University	14	82	188
2	Guangzhou University of Chinese Medicine	11	97	96
3	Chengdu University of Traditional Chinese Medicine	8	58	63
4	China Academy of Chinese Medical Sciences	5	37	66
5	Sun Yat-sen University	5	69	58
6	Tianjin University of Traditional Chinese Medicine	5	61	22
7	Huazhong University of Science & Technology	4	50	24
8	Shandong University of Traditional Chinese Medicine	4	29	42
9	Nanjing University of Chinese Medicine	3	12	22
10	Wuhan Hospital of Integrated Chinese & Western Medicine	2	32	25

### 3.4 Analysis of authors

**Figure 7:**
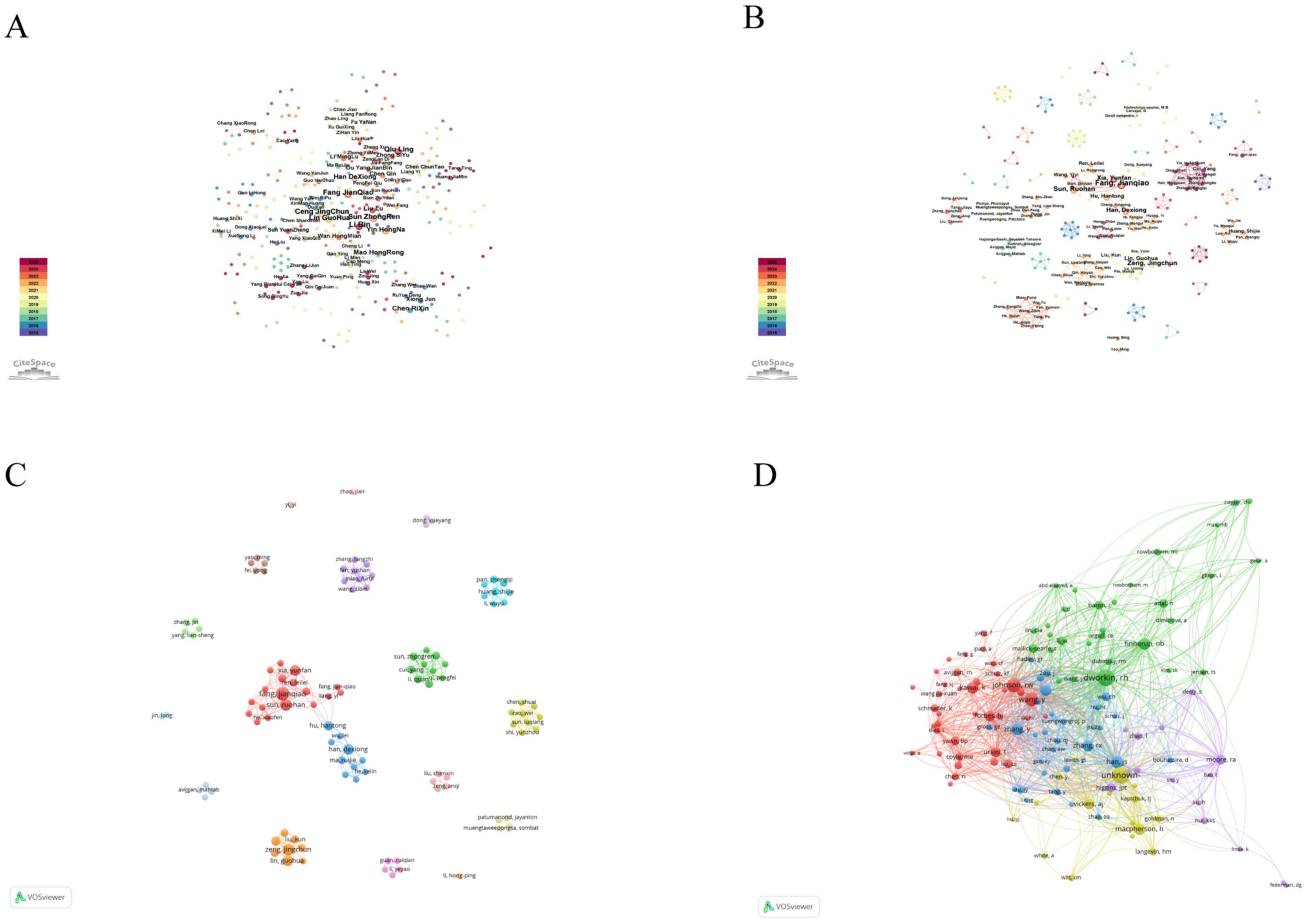
Author collaboration and co-citation analysis in acupuncture research on HZ. **(A)** Co-authorship network of authors from the Chinese databases. **(B)** Co-authorship network of authors from English databases. **(C)** Network visualization of author collaborations from the English databases. **(D)** Network visualization of co-cited authors from the English databases.

The distribution and influence of authors are illustrated in Figures 7A–D. In the Chinese database (Figure 7A), the leading authors were Li Bin (*n* = 12 publications), Fang Jianqiao (*n* = 10), Zeng Jingchun (*n* = 10), Sun Zhongren (*n* = 9), Lin Guohua (*n* = 9), and Qiu Ling (*n* = 9), followed by Yin Hongna, Chen Rixin, and Mao Hongrong (*n* = 8 each). In the English database (Figure 7B), Fang Jianqiao produced the largest number of publications (*n* = 8), followed by Zeng Jingchun (*n* = 6), Sun Ruohan (*n* = 6), Han Dexiong (*n* = 5), and Xia Yunfan (*n* = 5), with Chinese authors collectively contributing the majority of outputs (Table 4). Co-authorship network analysis using VOSviewer (Figure 7C) further identified Fang Jianqiao (*n* = 8 publications, 38 citations, total link strength = 40), Sun Ruohan (*n* = 6, 38, 35), Han Dexiong (*n* = 5, 28, 20), and Lin Guohua (*n* = 5, 72, 20) as the core authors with strong collaborative relationships, while emerging researchers such as Cui Yang, Li Quan, and Xia Yunfan showed moderate collaboration strength, forming secondary clusters within the network (Table 5). Co-citation analysis of cited authors (Figure 7D) revealed that Dworkin RH (*n* = 51 citations, total link strength = 775), Johnson RW (*n* = 36, 546), Wang Y (*n* = 34, 474), and Han JS (*n* = 26, 424) were the most frequently cited and academically influential authors, underscoring their central roles and substantial impact within the international research landscape (Table 6).

**Table 4:** Top 10 authors publishing research on acupuncture for HZ in Chinese and English databases.

Rank	Chinese database			English database		
	Author	Publications	Centrality	Author	Publications	Centrality
1	Li Bin	12	0	Fang, Jianqiao	8	0
2	Fang JianQiao	10	0	Zeng, Jingchun	6	0
3	Ceng JingChun	10	0	Sun, Ruohan	6	0
4	Sun ZhongRen	9	0	Han, Dexiong	5	0
5	Lin GuoHua	9	0	Xia, Yunfan	5	0
6	Qiu Ling	9	0	Lin, Guohua	4	0
7	Yin HongNa	8	0	Hu, Hantong	4	0.01
8	Chen RiXin	8	0	Ren, Leilei	3	0
9	Mao HongRong	8	0	Huang, Shijie	3	0
10	Han DeXiong	8	0	Wang, Yiyi	3	0

**Table 5:** Top 10 authors in co-authorship network for acupuncture research on HZ based on English databases.

Rank	Author	Publications	Citations	Total link strength
1	Fang Jianqiao	8	38	40
2	Sun Ruohan	6	38	35
3	Han Dexiong	5	28	20
4	Lin Guohua	5	72	20
5	Xia Yunfan	5	16	22
6	Cui Yang	3	10	22
7	Li Quan	3	10	22
8	Li Rongrong	3	23	15
9	Liu Kun	3	31	15
10	Lu Liming	3	66	14

**Table 6:** Top 10 cited authors in co-citation network for acupuncture research on HZ based on English databases.

Rank	Author	Citations	Total link strength
1	Dworkin RH	51	775
2	Johnson RW	36	546
3	Wang Y	34	474
4	Han JS	26	424
5	Vickers AJ	20	331
6	Finnerup NB	24	359
7	Zhang Y	24	430
8	Ursini T	17	254
9	Moore RA	17	495
10	Baron R	14	242

### 3.5 Analysis of journals

**Figure 8:**
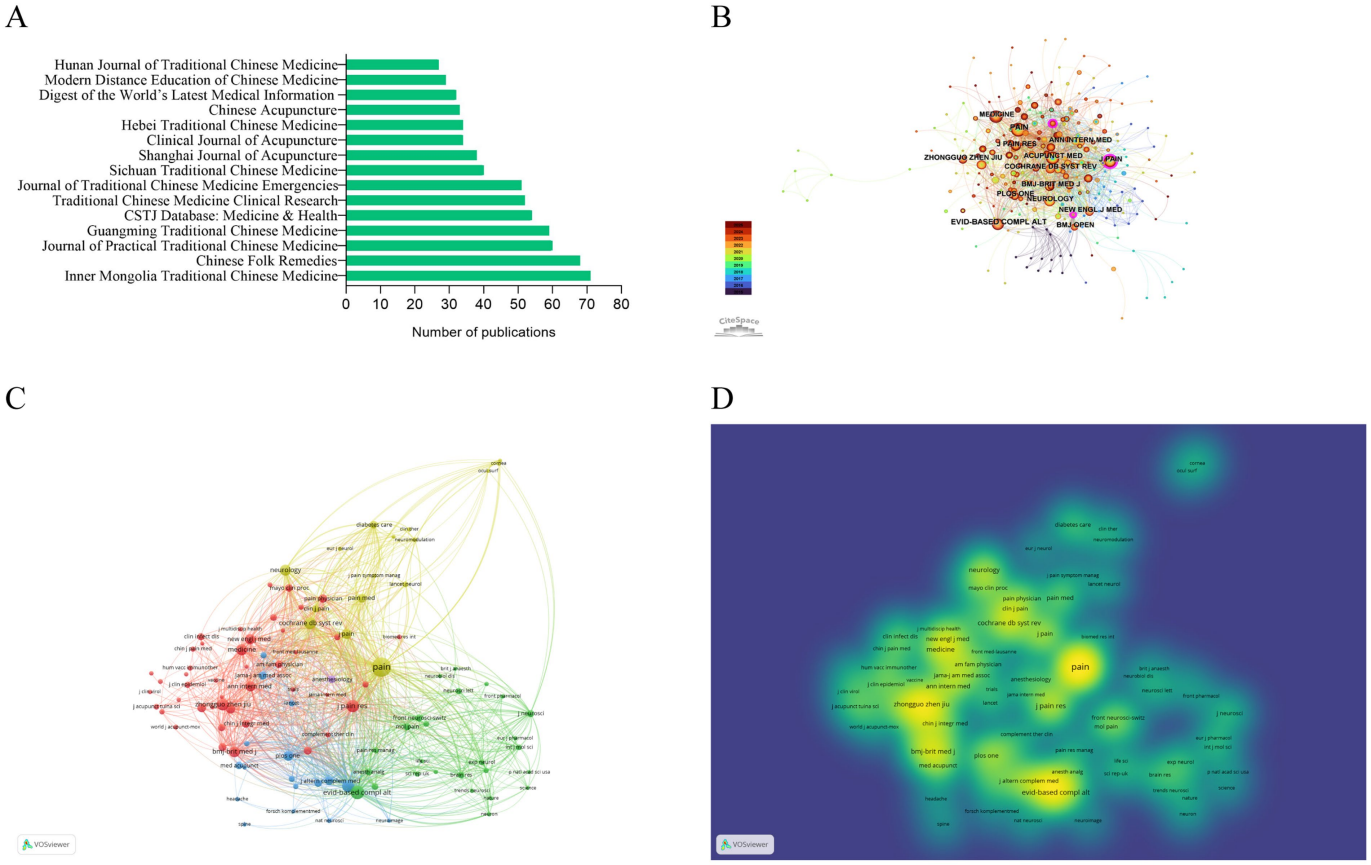
Journal distribution and co-citation analysis of acupuncture research on HZ. **(A)** Top 15 journals by publication volume in the Chinese databases. **(B)** Collaboration network of journals from the English databases. **(C)** Network visualization of co-cited journals from the English databases. **(D)** Density visualization of co-cited journals from the English databases.

The distribution and influence of journals are shown in Figure 8. In the Chinese database, the leading journals included *Inner Mongolia Traditional Chinese Medicine* (*n* = 71), *Chinese Folk Remedies* (*n* = 68), *Journal of Practical Traditional Chinese Medicine* (*n* = 60), and *Guangming Traditional Chinese Medicine* (*n* = 59) (Figure 8A). In the English database, *PAIN* (*n* = 62), *Evidence-Based Complementary and Alternative Medicine* (*n* = 50), *J Pain Res* (*n* = 45), and *Medicine* (*n* = 44) were the most prolific (Figure 8B and Table 7). Co-citation network analysis highlighted *PAIN* (214 citations, total link strength = 8,347), *Evidence-Based Complementary and Alternative Medicine* (104, 3,899), *J Pain Res* (86, 3,214), *BMJ-Brit Med J* (64, 2050), and *Zhongguo Zhen Jiu* (83, 1,522) as core journals with strong interconnections (Figure 8C and Table 8), while density visualization revealed high-impact clusters centered on these journals, alongside secondary clusters formed by other high-impact journals such as *Medicine* (69, 1849), *Cochrane Database of Systematic Reviews* (80, 2,915), *New Engl J Med* (58, 1835), *PLOS ONE* (56, 1881), and *Ann Intern Med* (57, 1804) (Figure 8D and Table 8), underscoring their central roles and influence in both clinical and research landscapes.

**Table 7:** Top 10 journals for publications on acupuncture research for HZ in English databases.

Rank	Journals	Publications	Centrality	Year
1	*PAIN*	62	0.07	2016
2	*Evidence-Based Complementary and Alternative Medicine*	50	0.06	2015
3	*J Pain Res*	45	0.04	2018
4	*Medicine*	44	0.10	2019
5	*Cochrane Database of Systematic Reviews*	41	0.04	2017
6	*BMJ-Brit Med J*	40	0.05	2017
7	*Zhongguo Zhen Jiu*	40	0.03	2015
8	*New Engl J Med*	39	0.03	2017
9	*PLOS ONE*	39	0.07	2015
10	*Ann Intern Med*	37	0.04	2017

**Table 8:** Top 10 journals in co-citation networks for acupuncture research on HZ based on English databases.

Rank	Journals	Citations	Total link strength
1	*PAIN*	214	8,347
2	*Evidence-Based Complementary and Alternative Medicine*	104	3,899
3	*J Pain Res*	86	3,214
4	*Medicine*	69	1849
5	*Cochrane Database of Systematic Reviews*	80	2,915
6	*BMJ-Brit Med J*	64	2050
7	*Zhongguo Zhen Jiu*	83	1,522
8	*New Engl J Med*	58	1835
9	*PLOS ONE*	56	1881
10	*Ann Intern Med*	57	1804

### 3.6 Analysis of references

**Figure 9:**
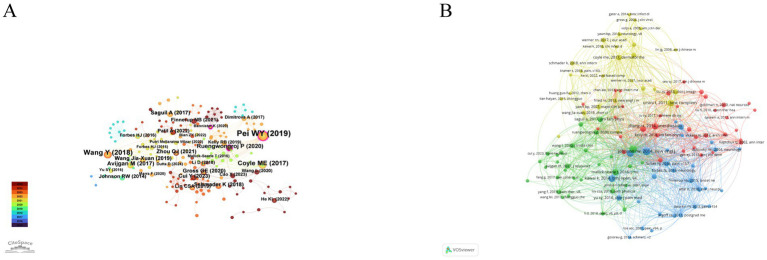
Analysis of cited references in acupuncture research on HZ from the English databases. **(A)** Citation network of references. **(B)** Network visualization of co-cited references.

Due to limitations in the Chinese database, reference analysis was performed using the English databases. The distribution and influence of cited references are shown in Figure 9. Reference citation analysis (Figure 9A and Table 9) identified *Pei WY (2019, J Pain Res)*, *Wang Y (2018, Medicine)*, and *Coyle ME (2017, Dermatol Ther)* as the most frequently cited studies, providing key evidence for the clinical efficacy of acupuncture in HZ. Highly central works such as *Ruengwongroj P (2020, Complement Ther Clin Pract)* and *Avijgan M (2017, J Acupunct Meridian Stud)* played bridging roles across research clusters, reflecting their influence on integrating clinical and mechanistic perspectives. Co-citation network of references (Figure 9B and Table 10) revealed that highly cited studies, such as Pei WY (2019, *J Pain Res*), Wang Y (2018, *Medicine*), and Coyle ME (2017, *Dermatol Ther*), occupied central positions and formed strong connections with other influential works. These central references highlight the foundational studies that integrate clinical evidence and mechanistic insights in acupuncture research for HZ.

**Table 9:** Top 10 cited references in citation networks for acupuncture research on HZ based on English databases.

Rank	References	Citations	Centrality	Year
1	Pei WY, 2019, *J Pain Res*	17	0.13	2019
2	Wang Y, 2018, *Medicine*	12	0.07	2018
3	Coyle ME, 2017, *Dermatol Ther*	9	0.03	2017
4	Ruengwongroj P, 2020, *Complement Ther Clin Pract*	7	0.18	2020
5	Avijgan M, 2017, *J Acupunct Meridian Stud*	7	0.08	2017
6	Schmader K, 2018, *Ann Intern Med*	6	0.01	2018
7	Wang Jia-Xuan, 2019, *Zhen Ci Yan Jiu*	6	0.05	2019
8	Zhou QJ, 2021, *Medicine*	6	0.05	2021
9	Cui Y, 2023, *Front Neurosci*	6	0.02	2023
10	Saguil A, 2017, *Am Fam Physician*	6	0.01	2017

**Table 10:** Top 10 co-cited references in acupuncture research on HZ based on English databases.

Rank	Co-cited reference	Citations	Total link strength
1	Pei WY, 2019, *J Pain Res*,	21	278
2	Wang Y, 2018, *Medicine*	17	227
3	Coyle ME, 2017, *Dermatol Ther*	12	129
4	Ruengwongroj P, 2020, *Complement Ther Clin Pract*	8	116
5	Avijgan M, 2017, *J Acupunct Meridian Stud*	10	61
6	Vickers AJ, 2012, *Arch Intern Med*	8	71
7	Wu CH, 2013, *Mol Pain*,	13	141
8	Zhang RX, 2014, *Anesthesiology*	15	166
9	Wang Jia-Xuan, 2019, *Zhen Ci Yan Jiu*	8	78
10	Zhou QJ, 2021, *Medicine*	8	105

### 3.7 Analysis of keywords

**Figure 10:**
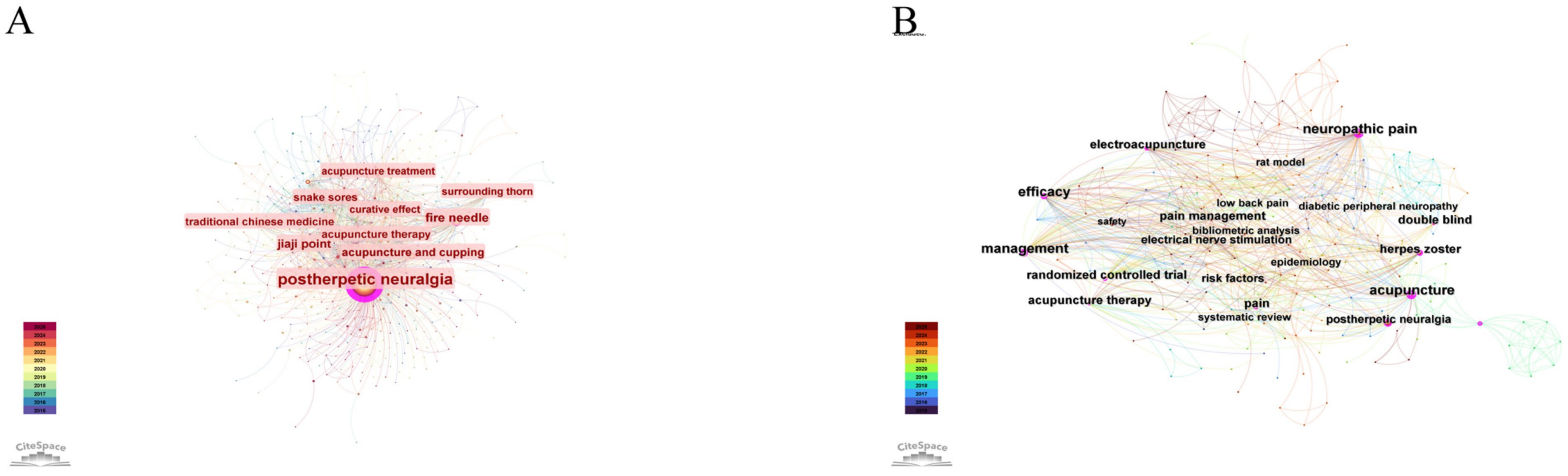
Keyword co-occurrence analysis of acupuncture research on HZ. **(A)** Co-occurrence network of keywords from the Chinese database. **(B)** Co-occurrence network of keywords from the English database.

CiteSpace analysis of keyword co-occurrence revealed the most frequent and central topics in acupuncture research on HZ across both databases (Table 11 and Figure 10). In the Chinese database, PHN (*n* = 860, centrality = 1.10), acupuncture and cupping (145, 0.06), fire needle (132, 0.14), TCM (89, 0.06), and Jiaji point (86, 0.08) were the top keywords, with PHN showing the highest centrality. In the English database, HZ (*n* = 59, 0.17), PHN (57, 0.13), management (23, 0.26), neuropathic pain (23, 0.35), and acupuncture (20, 0.40) predominated, with acupuncture and neuropathic pain demonstrating the highest centrality, highlighting their pivotal role in structuring research networks across both Chinese and English literature.

**Table 11:** Top 10 keywords in acupuncture research on HZ in Chinese and English databases.

Rank	Chinese database			English databases		
	Keywords	Count	Centrality	Keywords	Count	Centrality
1	Postherpetic Neuralgia	860	1.10	Herpes Zoster	59	0.17
2	Acupuncture and Cupping	145	0.06	Postherpetic Neuralgia	57	0.13
3	Fire Needle	132	0.14	Management	23	0.26
4	Traditional Chinese Medicine	89	0.06	Neuropathic Pain	23	0.35
5	Jiaji Point	86	0.08	Acupuncture	20	0.40
6	Acupuncture Therapy	84	0.05	Pain	16	0.11
7	Snake Sores	74	0.06	Electroacupuncture	12	0.12
8	Clinical Efficacy	69	0.03	Efficacy	11	0.24
9	Acupuncture Treatment	63	0.05	Acupuncture Therapy	10	0.13
10	Curative Effect	62	0.05	Randomized Controlled Trial	10	0.11

**Figure 11:**
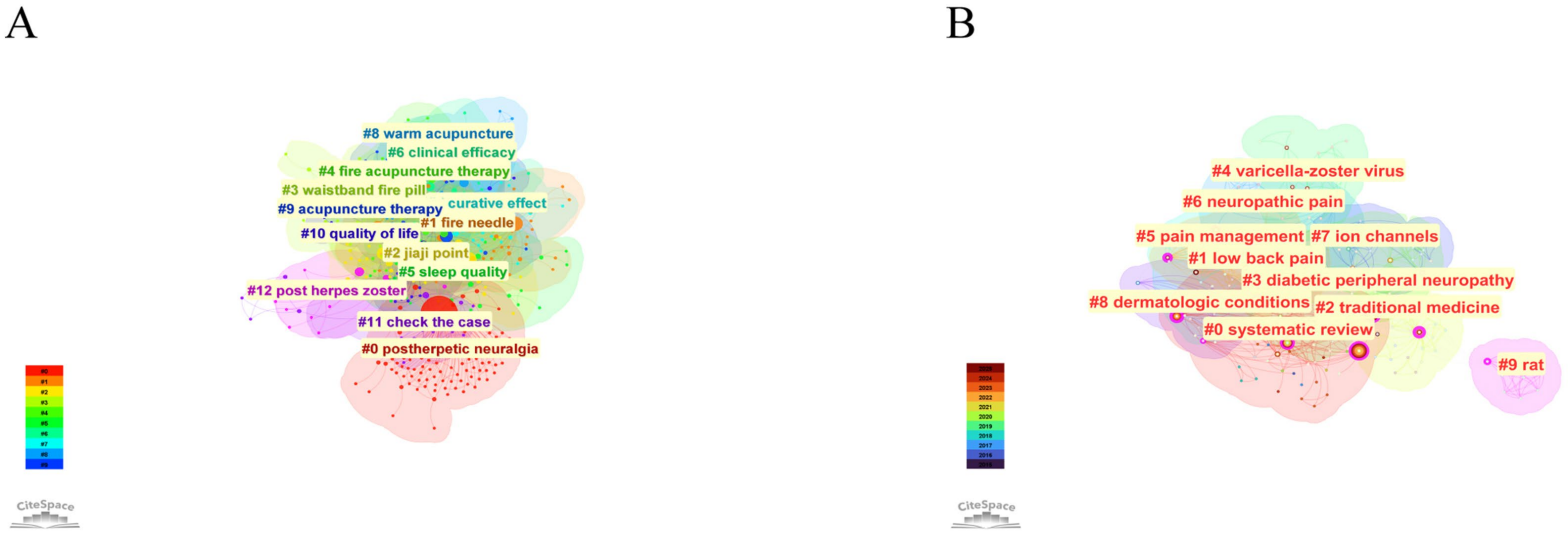
Clustered keyword analysis of acupuncture research on HZ. **(A)** Clustered co-occurrence network of keywords from the Chinese database. **(B)** Clustered co-occurrence network of keywords from the English database.

Clustered network analysis, which summarizes frequently co-occurring keywords through computational methods, was performed using CiteSpace, with silhouette scores above 0.7 indicating valid and reliable clusters. In the Chinese literature (Figure 11A), the main clusters were PHN (#0), fire needle (#1), jiaji point (#2), waistband fire pill (#3), fine acupuncture therapy (#4), sleep quality (#5), clinical efficacy (#6), curative effect (#7), warm acupuncture (#8), acupuncture therapy (#9), quality of life (#10), and case assessment (#11), with a modularity of *Q* = 0.5373 and a weighted mean silhouette of S = 0.8684. In the English literature (Figure 11B), the prominent clusters included systematic review (#0), low back pain (#1), traditional medicine (#2), diabetic peripheral neuropathy (#3), VZV (#4), pain management (#5), neuropathic pain (#6), ion channels (#7), dermatologic conditions (#8), and rat model (#9), showing higher modularity (*Q* = 0.5794) and a perfect silhouette value (*S* = 1.0). These results suggest that the Chinese literature predominantly focuses on the clinical application and efficacy of acupuncture and herbal therapies for PHN whereas the English literature emphasizes broader scientific themes, including evidence synthesis, underlying mechanisms, and diverse neuropathic pain conditions.

**Figure 12:**
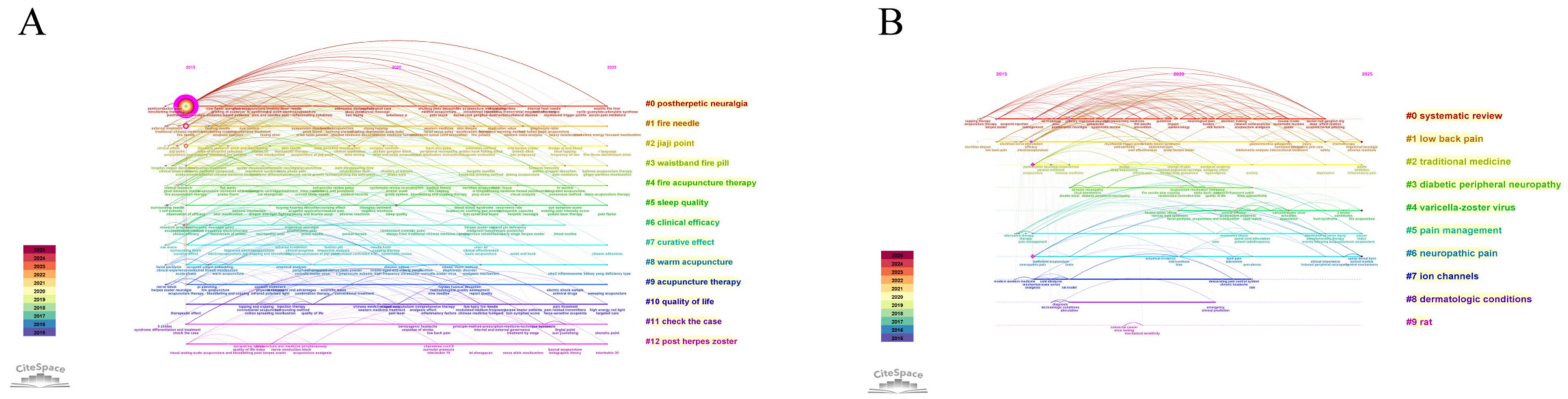
Timeline analysis of keyword evolution in acupuncture research on HZ. **(A)** Temporal evolution of co-occurring keywords in the Chinese database. **(B)** Temporal evolution of co-occurring keywords in the English database.

Timeline analysis using CiteSpace captured the temporal evolution of co-occurring keywords. In the Chinese literature (Figure 12A), early research (2015–2018) focused on PHN, basic acupuncture techniques (e.g., fire needle, jiaji point), and disease-specific interventions. From 2019 to 2022, studies expanded to mechanisms, integrated therapies, and patient-reported outcomes such as sleep quality and quality of life. Recent work (2023–2025) emphasized precision interventions and innovative techniques. In the English literature (Figure 12B), initial research (2015–2018) concentrated on traditional therapies and basic mechanisms (ion channels, chronic pain models). Between 2019 and 2022, the focus shifted to interdisciplinary integration, systematic reviews, and mechanism exploration, while 2023–2025 studies highlighted precise mechanistic insights, clinical translation, and experimental models. Overall, the Chinese timeline reflects disease-specific, technique-driven clinical research, whereas the English timeline shows broader chronic pain topics, mechanism studies, and evidence-based approaches.

**Figure 13:**
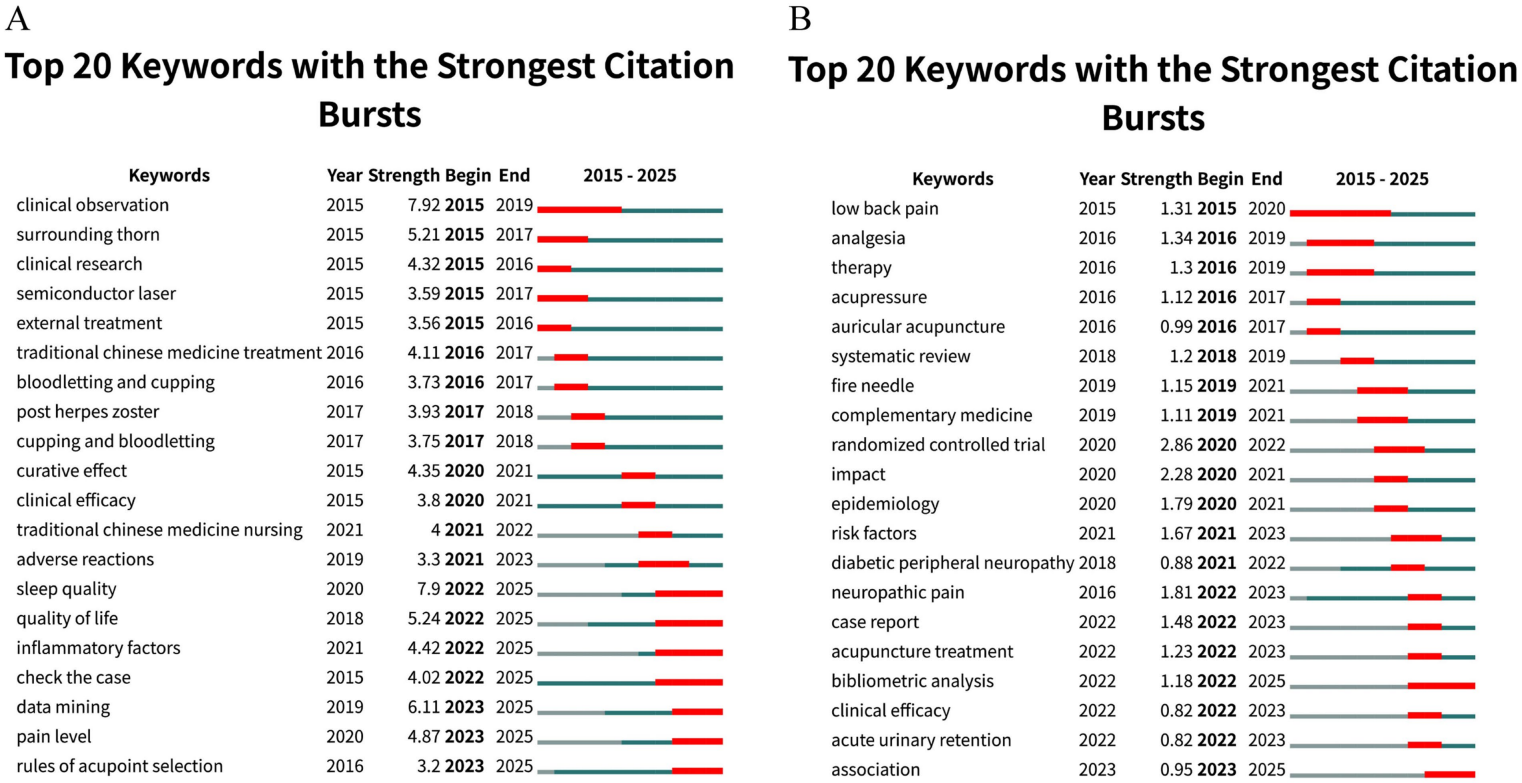
Keyword citation burst analysis in acupuncture research on HZ. **(A)** Citation bursts of keywords in the Chinese database. **(B)** Citation bursts of keywords in the English database.

Keyword citation burst analysis (Figure 13) illustrates the temporal evolution of research hotspots. In the Chinese literature (Figure 13A), early bursts (2015–2017) centered on TCM external therapies and preliminary clinical studies, such as fire needle, cupping, and clinical observation. Between 2017 and 2021, focus shifted to PHN, clinical efficacy, curative effect, and TCM nursing. From 2021 to 2025, bursts emphasized inflammatory factors, sleep quality, quality of life, acupoint selection, and case analysis, reflecting mechanistic exploration, patient-centered outcomes, and refinement of techniques. In the English literature (Figure 13B), early bursts (2015–2018) highlighted core diseases and evidence-based methods, including low back pain, neuropathic pain, systematic reviews, and randomized controlled trials (RCTs). Between 2019 and 2021, research expanded to mechanisms, risk factors, and epidemiology, while 2022–2025 saw bursts in bibliometric analysis, acute urinary retention, case reports, and association studies. Overall, the Chinese data show a progression from TCM techniques to disease-specific efficacy, then to mechanisms and patient outcomes, whereas the English data show a trajectory from disease focus to mechanistic and epidemiological insights, then to clinical precision and field-level evaluation.

## 4 Discussion

### 4.1 General information

This bibliometric analysis provides a comprehensive overview of global research on acupuncture for HZ, drawing on publications from both Chinese and English databases in recent years. A notable disparity exists between the number of Chinese (*n* = 2,165) and English (*n* = 144) publications, which may disproportionately influence the overall “global” picture presented. This imbalance suggests that trends, collaborative networks, and identified hotspots are largely shaped by domestic Chinese literature, while insights from international studies may be underrepresented. Annual publication trends reveal distinct patterns between domestic and international literature. Chinese publications reached their highest number in 2018 and continued to maintain a substantial output thereafter, suggesting continued domestic focus and active research efforts. In contrast, English literature demonstrated a steady upward trajectory, surpassing 20 publications for the first time in 2022, suggesting growing international recognition and academic engagement in this research domain. This divergence in publication trends between Chinese and international literature is a common phenomenon observed in region-specific medical research, reflecting differing research ecosystems and dissemination priorities (18).

At the country level, China dominated in terms of publication output, followed by the United States, Australia, and South Korea. Betweenness centrality analysis identified China and the United States as central hubs in the global collaboration network, while collaborations among other countries remain limited. This finding aligns with broader trends in complementary medicine research, where China leads in output while the United States often serves as a central node in international collaboration networks (19). At the institutional level, Zhejiang Chinese Medical University, Guangzhou University of Chinese Medicine, and Chengdu University of Traditional Chinese Medicine emerged as the most productive and influential institutions, highlighting the pivotal role of Chinese institutions in driving research. Co-authorship and citation network analyses revealed that established researchers form the backbone of domestic collaborations, whereas emerging authors participate in secondary clusters, indicating a gradually expanding collaborative network characteristic of a developing research field (20).

High-impact journals, including *PAIN*, *Evidence-Based Complementary and Alternative Medicine*, and *J Pain Res*, serve as primary outlets for influential research, while Chinese journals predominantly focus on clinical case reports and TCM-specific therapies. The prominence of these international journals underscores the field’s ongoing integration into mainstream pain and evidence-based medicine discourse (21). Co-citation analysis identified foundational authors such as Dworkin RH, Johnson RW, Wang Y, and Han JS, whose works have shaped the intellectual structure and research directions of the field. Although these co-cited studies are influential, many appear to involve relatively small sample sizes, variable methodological reporting, or descriptive designs, suggesting that the strength of evidence may be limited.

### 4.2 Hot spots and trends

Keyword co-occurrence and clustering analyses reveal evolving research priorities. In the Chinese literature, the most frequent and central keywords — PHN, acupuncture and cupping, fire needle, TCM, and Jiaji Point — reflect the core clinical and research focus of the field (18). However, given the dominance of Chinese publications, the prominence of technique-specific keywords (e.g., fire needle, Jiaji Point) may primarily reflect domestic research priorities rather than global trends. Consequently, identified “hot topics” should be interpreted with caution, recognizing that mechanism-oriented or interdisciplinary research highlighted in English publications may be underrepresented in the overall analysis. This focus aligns with findings from a bibliometric analysis of acupuncture for pain conditions, which identified “PHN” and “neuropathic pain” as central themes in the Chinese research landscape (22). Early cluster analyses (2015–2018) primarily emphasized traditional acupuncture techniques, such as fire needle, Jiaji points, and warm acupuncture, which have demonstrated efficacy in alleviating neuropathic pain and improving local circulation (23, 24). For example, the application of fire needle at Jiaji points has been shown to rapidly reduce PHN pain scores while improving local skin perfusion, supporting its continued clinical use (25). The subsequent shift in keyword focus toward inflammatory markers, immune modulation, and patient-reported outcomes from 2019 onwards reflects a maturation of the field—from descriptive studies emphasizing procedural techniques to more mechanistic and evidence-based investigations. This evolution indicates that researchers are increasingly probing the biological underpinnings of acupuncture analgesia, integrating clinical observation with molecular and immunological insights, thereby enhancing the scientific rigor and translational relevance of the field.

From 2019 to 2022, research shifted toward integrative approaches and patient-centered outcomes, including sleep quality, functional recovery, and overall quality of life (26, 27). For instance, combining fire needle therapy with cupping or electroacupuncture has been reported to provide superior pain relief while enhancing sleep efficiency and psychological well-being in PHN patients (25). Similarly, integrating acupuncture with moxibustion or pharmacological therapy has demonstrated additive effects on reducing pain intensity and improving quality-of-life scores (28), highlighting the value of multimodal treatment strategies. More recent publications (2023–2025) have emphasized precision interventions and novel acupuncture modalities, reflecting a transition from symptom-focused treatment to mechanism-guided, individualized therapy. For example, studies indicate that targeting specific acupoints while adjusting stimulation parameters based on patient characteristics can modulate immune cell balance (Th17/Treg) and neurotransmitter pathways, providing a biological rationale for personalized treatment approaches (29, 30). These trends suggest a growing interest in mechanistic research; however, many studies involve small sample sizes, single-center designs, or preliminary mechanistic exploration, indicating that high-quality confirmatory evidence is still limited. This trend towards mechanistic exploration is consistent with the broader evolution of acupuncture research, which is increasingly interrogating its neuro-immunological foundations, as highlighted in foundational reviews of acupuncture analgesia (31).

In the English-language literature, keyword clusters highlighted systematic reviews, neuropathic pain, diabetic peripheral neuropathy, low back pain, and pain management. The prominence of “systematic review” and the inclusion of broader pain conditions like “low back pain” and “diabetic peripheral neuropathy” are characteristic of international acupuncture research, which often situates the therapy within the wider context of chronic neuropathic pain management, a pattern also observed in other scientometric studies (32, 33). While systematic reviews and meta-analyses are frequently cited, the conclusions generally suggest positive but limited evidence due to heterogeneity of interventions, small sample sizes, and variable methodological quality. This indicates potential evidence gaps despite the recognition of these reviews as hotspots in the field. Temporal trajectory analysis reveals a progression from mechanistic investigations and traditional therapies (2015–2018), through evidence synthesis and interdisciplinary integration (2019–2022), to the most recent phase (2023–2025) with translational studies, precise mechanistic evaluations, and experimental models. For example, systematic reviews and meta-analyses have helped clarify the comparative effectiveness of different acupuncture techniques, guiding evidence-based clinical decisions for PHN and related neuropathic pain conditions (34). The high-impact systematic review by Pei et al. (35), identified in our co-citation analysis, is a cornerstone of this evidence-synthesis phase, having significantly shaped clinical understanding by consolidating evidence on acupuncture’s efficacy for PHN. Nevertheless, most reviews highlight limitations in the primary studies, such as small RCTs, lack of blinding, and heterogeneity of protocols, underscoring the need for higher-quality trials to confirm clinical efficacy. These patterns indicate that international research increasingly prioritizes clinically translatable evidence while exploring underlying neuroimmunological and molecular mechanisms, but the overall maturity of the evidence base remains developing.

Citation-burst analyses further illustrate these temporal dynamics. In Chinese publications, early bursts focused on TCM external therapies, mid-period bursts emphasized PHN management and clinical efficacy, and recent bursts highlight inflammatory markers, acupoint selection, and patient-reported outcomes, reflecting growing interest in mechanistic and patient-centered research (36). For example, recent studies suggest that careful selection of Ashi and Jiaji points, combined with adjuvant therapies like cupping or mild moxibustion, can optimize pain relief and sleep quality, providing practical guidance for clinicians (30, 37). However, these studies are often small-scale and single-center, limiting generalizability. In English publications, early bursts were disease-focused, mid-period bursts addressed mechanistic pathways and epidemiology, and recent bursts reflect methodological diversification, including bibliometric studies and case reports. The early, high-impact clinical and epidemiological studies by authors like Johnson RW and Dworkin RH, which were among the most cited references in our analysis, laid the essential groundwork for defining PHN and its burden, thereby framing the clinical problem that subsequent acupuncture research aimed to address (38). Across both languages, burst patterns highlight influential topics but also reveal that rigorous, large-scale RCT evidence remains limited, indicating a gap between research activity and high-certainty evidence.

Collectively, these findings suggest complementary yet distinct trends between domestic and international research. Chinese studies emphasize clinical application, TCM-specific techniques, and patient outcomes, whereas international studies prioritize methodological rigor, mechanistic insight, and evidence synthesis. This observed dichotomy echoes the conclusions of a comparative bibliometric study on TCM research output, which noted a stronger orientation towards pragmatic trials and technique refinement in domestic Chinese literature compared to a more mechanistic and critical appraisal-focused approach in international publications (38). Integrating these perspectives—combining clinical efficacy, mechanistic understanding, and robust methodology—may accelerate the development of precision acupuncture interventions and enhance the management of PHN. While some critiques question the biological plausibility of acupuncture points (4, 39), our findings, particularly the emerging link between specific acupoints (e.g., Jiaji) and measurable immunomodulatory outcomes, provide a compelling counterpoint and a pathway for resolving such debates through rigorous science. For instance, translating mechanistic insights from experimental studies into individualized clinical protocols could optimize patient-centered outcomes while maintaining high evidence standards. This integrative and translational approach is increasingly recognized as the future direction for validating and optimizing complex interventions like acupuncture.

### 4.3 Advantages and shortcomings

This bibliometric study provides a systematic overview of acupuncture research on HZ, integrating data from both Chinese and English databases. In contrast to the previous analysis by Chen et al. (18), which focused primarily on publication trends and keyword co-occurrence, the present study incorporates VOSviewer-based visualization, as well as analyses of country-level contributions, journal productivity, and co-citation networks. These additions enable a more comprehensive mapping of the research landscape, revealing influential authors, institutions, and journals, and offering deeper insights into the intellectual structure and evolving hotspots, including the transition from traditional acupuncture techniques toward integrative, mechanistic, and patient-centered approaches.

Nevertheless, several limitations should be acknowledged. Despite extensive database coverage, some relevant studies may have been missed, and language bias could have influenced the results. The study period was set from January 1, 2015, to October 1, 2025, to provide an updated overview of the most recent trends in this field. However, the inclusion of only partial data for 2025 may slightly bias the depiction of publication trends and emerging hotspots for that year. The analysis is primarily quantitative and does not include a formal assessment of methodological rigor, study design quality, or risk of bias, limiting inferences regarding the strength of the evidence. International collaboration remains relatively limited, with the majority of publications originating from China and a few high-output countries. Additionally, emerging mechanistic studies and recent innovations may be underrepresented due to low citation counts. Additionally, emerging mechanistic studies and recent innovations may be underrepresented due to low citation counts or small-scale design, reinforcing the need for further rigorous research to strengthen the evidence base.

## 5 Conclusion

This bibliometric study, integrating data from both Chinese and English databases through CiteSpace and VOSviewer, provides a comprehensive overview of acupuncture research on HZ and PHN. The findings reveal that research has progressively evolved from traditional acupuncture techniques toward integrative, mechanism-based, and patient-centered approaches. Chinese scholars have contributed the majority of publications, emphasizing clinical applications and traditional modalities, while international studies increasingly focus on methodological rigor, mechanistic exploration, and evidence synthesis.

Despite these advances, several gaps remain. Cross-regional collaboration and high-quality multicenter studies are still limited, and mechanistic investigations linking acupuncture to neuroimmune and pain modulation pathways require further development. Future research should strengthen international cooperation, conduct large-scale RCTs, and adopt standardized reporting protocols to enhance reproducibility and comparability. By integrating clinical efficacy with mechanistic insights, acupuncture research can further contribute to precision and evidence-based management of HZ and PHN.

## Data Availability

The original contributions presented in the study are included in the article/supplementary material, further inquiries can be directed to the corresponding author.
